# Supplementary material for: Wastewater Metagenomic Virome Analysis of the 2026 World Cup in Texas
Source: medRxiv. 2026 Sep 10:2026.09.04.26362178. Preprint. [Version 1] doi: 10.64898/2026.09.04.26362178 (PMC13622730; doi:10.64898/2026.09.04.26362178)
Supplement: Supplementary file 1 [file NIHPP2026.09.04.26362178v1-supplement-1.pdf]

medRxiv preprint doi: https://doi.org/10.64898/2026.09.04.26362178; this version posted September 10, 2026. The copyright holder for this preprint (which was not certified by peer review) is the author/funder, who has granted medRxiv a license to display the preprint in perpetuity.

It is made available under a CC-BY 4.0 International license .

Supplemental Material

Supplemental Figure 1: Weekly viral RPKMF signal by respiratory viral strain at each of the six historic sites in Houston spanning 2022 to 2026.

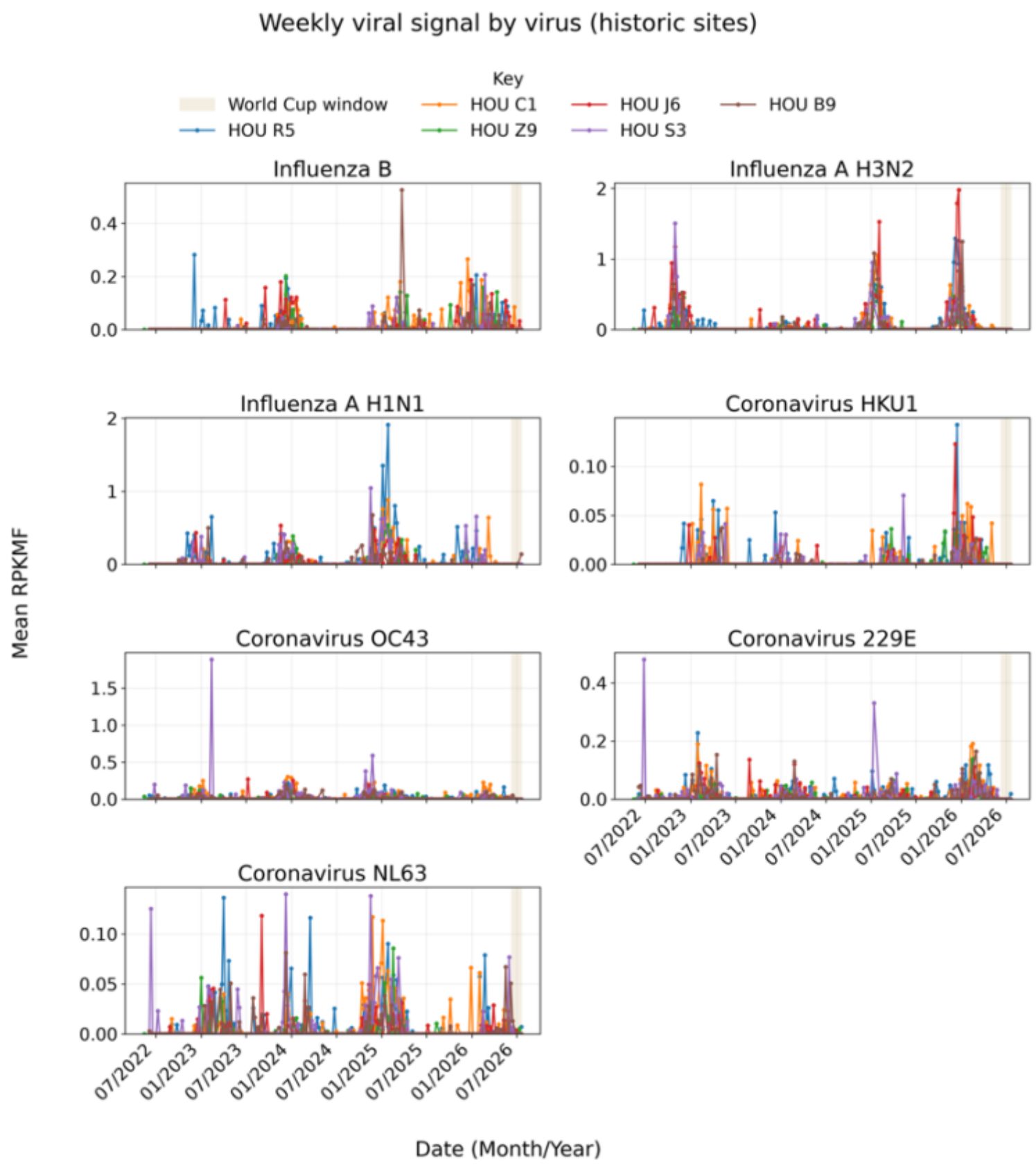

Figure S1 | Weekly viral abundance of respiratory viruses at each of the six historic sites in Houston spanning from 2022 to 2026. Time-series plots of the seven seasonal viral abundances (RPKMF) per week since the start of the TexWEB program. Each color represents a specific Houston site, and the shaded band marks the tournament window.

medRxiv preprint doi: https://doi.org/10.64898/2026.09.04.26362178; this version posted September 10, 2026. The copyright holder for this preprint (which was not certified by peer review) is the author/funder, who has granted medRxiv a license to display the preprint in perpetuity.

It is made available under a CC-BY 4.0 International license .

Supplemental Figure 2: Rare-taxon load over time between host and non-host cities at 3, 5, & 20 thresholds.

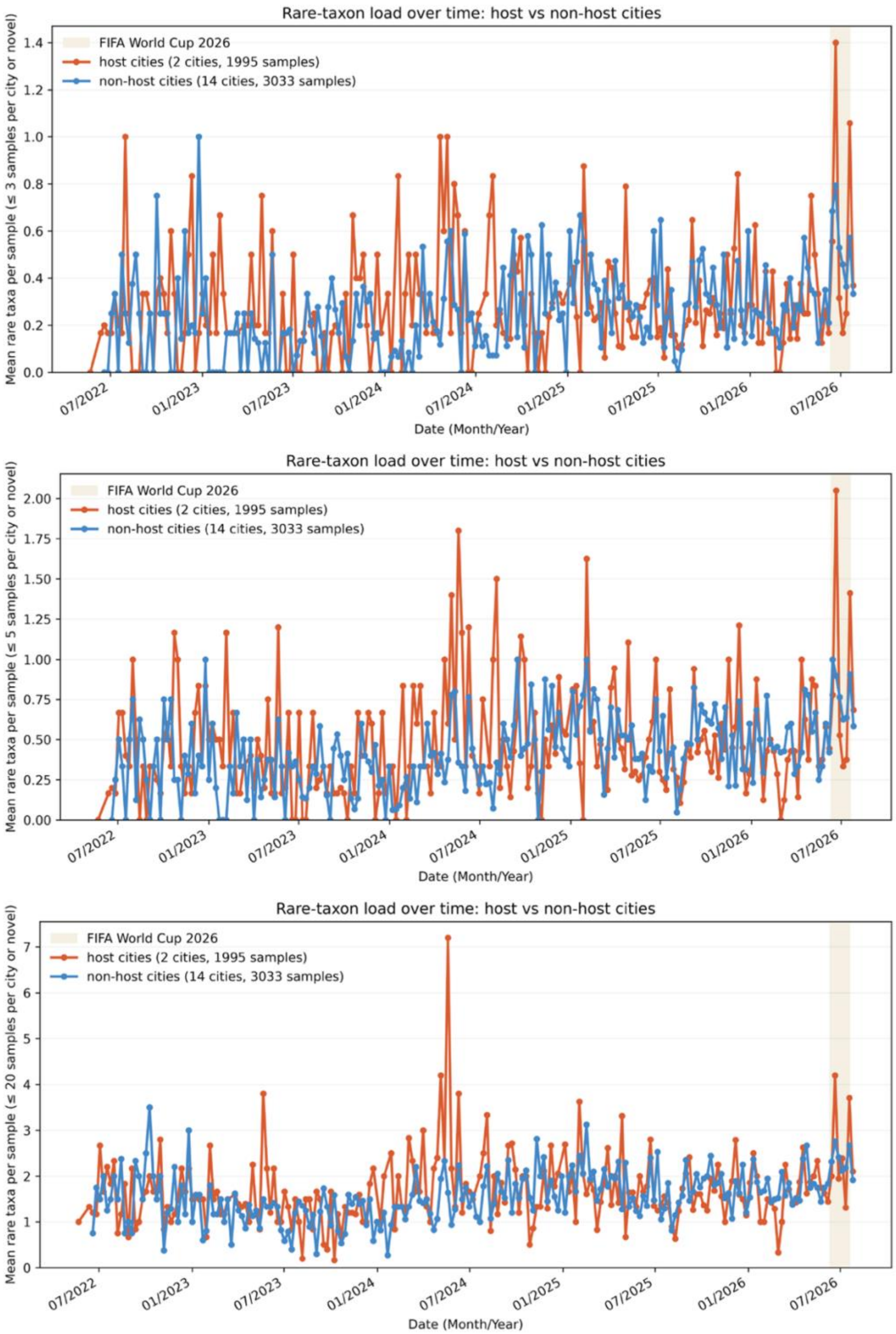

Figure S2 | Time-series plot of rare-taxon load between host and non-host cities at thresholds of 3, 5, and 20 viruses. Weekly mean count of unusual taxa per sample, at thresholds of 3 viruses for the top panel, 5 viruses at the center panel, and 20 viruses for the bottom panel. Unusual taxa represents both rare and novel taxa during the world cup tournament window. The shaded band marks the 2026 FIFA World Cup window.

medRxiv preprint doi: https://doi.org/10.64898/2026.09.04.26362178; this version posted September 10, 2026. The copyright holder for this preprint (which was not certified by peer review) is the author/funder, who has granted medRxiv a license to display the preprint in perpetuity.

It is made available under a CC-BY 4.0 International license .

Supplemental Table 1: Per-site Mann-Whitney U-test comparison of Summer 2026-vs-historical against historical-vs-historical median Bray-Curtis dissimilarity.

Supplemental Table 2: Mann-Whitney U test for rare-taxa per sample between host and non-host cities during the tournament window.

Supplemental Table 3: Mann-Whitney U test of Summer 2026 rare-taxa load to prior summers.

Supplemental Table 4: List of novel taxa sampled during the World Cup tournament sampling window.
